# Impact of weight trajectory after bariatric surgery on co-morbidity evolution and burden

**DOI:** 10.1186/s12913-020-5042-9

**Published:** 2020-04-03

**Authors:** Jason A. Davis, Rhodri Saunders

**Affiliations:** Coreva Scientific GmbH & Co KG, Im Muehlenbruch 1, 3rd Floor, 53639 Koenigswinter, Germany

**Keywords:** Weight-loss trajectory, Burden, Costs, Bariatric surgery, Gastric bypass, Diabetes, Hypertension

## Abstract

**Background:**

Bariatric surgery, such as Roux-en-Y gastric bypass [RYGB] has been shown to be an effective intervention for weight management in select patients. After surgery, different patients respond differently even to the same surgery and have differing weight-change trajectories. The present analysis explores how improving a patient’s post-surgical weight change could impact co-morbidity prevalence, treatment and associated costs in the Canadian setting.

**Methods:**

Published data were used to derive statistical models to predict weight loss and co-morbidity evolution after RYGB. Burden in the form of patient-years of co-morbidity treatment and associated costs was estimated for a 100-patient cohort on one of 6 weight trajectories, and for real-world simulations of mixed patient cohorts where patients experience multiple weight loss outcomes over a 10-year time horizon after RYGB surgery. Costs (2018 Canadian dollars) were considered from the Canadian public payer perspective for diabetes, hypertension and dyslipidaemia. Robustness of results was assessed using probabilistic sensitivity analyses using the R language.

**Results:**

Models fitted to patient data for total weight loss and co-morbidity evolution (resolution and new onset) demonstrated good fitting. Improvement of 100 patients from the worst to the best weight loss trajectory was associated with a 50% reduction in 10-year co-morbidity treatment costs, decreasing to a 27% reduction for an intermediate improvement. Results applied to mixed trajectory cohorts revealed that broad improvements by one trajectory group for all patients were associated with 602, 1710 and 966 patient-years of treatment of type 2 diabetes, hypertension and dyslipidaemia respectively in Ontario, the province of highest RYGB volume, corresponding to a cost difference of $3.9 million.

**Conclusions:**

Post-surgical weight trajectory, even for patients receiving the same surgery, can have a considerable impact on subsequent co-morbidity burden. Given the potential for alleviated burden associated with improving patient trajectory after RYGB, health care systems may wish to consider investments based on local needs and available resources to ensure that more patients achieve a good long-term weight trajectory.

## Background

Worldwide, obesity and its management are growing healthcare concerns. Canada faces similar issues with an increasing burden of obesity and related co-morbidities [1]. Being overweight or obese considerably increases the risk of developing diseases such as type 2 diabetes (T2D), hypertension (HTN), dyslipidaemia (DLP), or other forms of cardiovascular disease such as stroke or coronary artery disease. Managing these co-morbidities creates considerable burden for public and private payers in terms of hospital resource usage, physician visits and medications. Considering the chronic diseases most commonly associated with obesity, for example, costs of care in Canada were estimated to have increased by 19% between 2000 and 2008, from $3.9 to $4.6 billion [2].

The ideal treatment must be optimised individually between patient and provider and involves a long-term commitment to improvement of health. Some patients may be supported with diet and exercise, some with medication, and for many patients, a key catalyst to support these lifelong changes is bariatric surgery. Procedures performed in Canada include established surgeries such as Roux-en-Y gastric bypass (RYGB), sleeve gastrectomy, biliopancreatic diversion with or without duodenal switch and adjustable gastric banding, although the latter is decreasing in use [3, 4]. The choice of surgery will impact the potential range of weight loss a patient may achieve and degree to which co-morbidities are resolved or improved [5–9].

Among patients who do receive surgery, there is a wide range of outcomes for weight loss and co-morbidity resolution. Differences occur between surgery types, and within the same surgical cohort, where some patients experience good trajectories with considerable early and sustained weight loss with concomitant co-morbidity resolution, while others follow poorer trajectories of lower weight loss and persistent co-morbidities [10, 11]. The latter, poorer weight loss may result in insufficient improvement in weight status to reduce co-morbidity risks, both extending the risks to patient health and maintaining a high burden of care for payers.

The reasons for different weight trajectories after the same type of surgery remain to be elucidated. Factors such as sex or gender, race and genetics have been investigated, [10–12] but with an aim to improving surgical outcomes, these characteristics are not readily modifiable parameters and clinical application of these contributors remains to be determined [12]. Other interventions may yet be possible, however, given the observation that early post-surgical results during the time of greatest patient contact have been shown to be a reliable predictor of longer term results [10] and a retrospective audit identified missing post-surgical clinical attendance as a main predictor of poor long-term weight loss [13].

To date, analyses of the burden of obesity have focused on bariatric surgery versus medical management, or between different types of surgery. The variation in outcomes within a single type of surgery could, however, also be a substantial cost driver. The aim of this study is to provide a first estimate quantifying the differences in patient outcomes and costs between patients following different weight trajectories in the Canadian setting after one form of bariatric surgery - RYGB.

## Methods

**Fig. 1:**
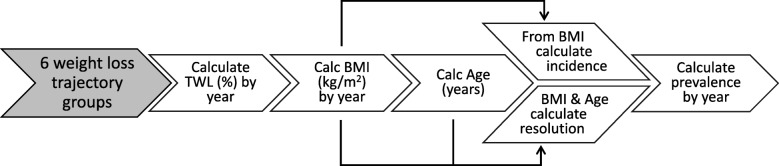
Scheme for analysis of outcome and cost impact by weight trajectory. The above schematic depicts the sequence of calculations performed separately for each of 6 weight loss trajectory groups as defined in the study of Courcoulas et al. Percent total weight loss (%TWL) is modelled as a function of time over the 10-year time horizon post-RYGB. From the patient cohort demographics, the modelled TWL is converted to body mass index (BMI) for each post-surgical year in the two trajectories and time after surgery converted to age. The yearly BMI is used to calculate the incidence of new co-morbidity cases in patients without the co-morbidity at baseline, while the yearly BMI and patient age are used to calculate the resolution in patients who had the co-morbidity at baseline. Together, these are used to calculate the annual prevalence. Summing over the time horizon, the impact on cases to be treated is taken as the difference between cases in the poor trajectory minus those in the good trajectory. This difference is multiplied by annual co-morbidity treatment costs to determine the impact on costs

Here, a weight trajectory is the evolution of patient weight after bariatric surgery. In the present analysis, good weight trajectories are taken as those that demonstrate considerable early weight loss that is maintained in the mid- to long-term, while poorer trajectories show lesser initial weight gain and either greater weight regain after a nadir, or sustained low weight loss. A recent study by Courcoulas et al. [10] (1738 patients who underwent RYGB, 7 years follow-up) determined 6 trajectories into which the patients’ post-surgical weight loss outcomes could be grouped. Data were further presented regarding the evolution of the patients’ co-morbidities stratified by weight loss trajectory group, thereby providing a first detailed assessment of the association between separate weight loss outcomes and co-morbidity burden within the same surgical cohort. Applying these data in the present study, the prevalence of three co-morbidities (T2D, HTN, and DLP as high low density lipoprotein as reported by Courcoulas et al) could be estimated as a function of post-RYGB weight trajectory by considering both resolution in patients with the co-morbidity at baseline and new onset among those without. Models were employed, informed by available data, to extrapolate reported outcome results to a 10-year window. The analysis scheme is depicted in Fig. 1.

### Study cohort

The main analyses in the present study consider a cohort of 100 patients who have undergone RYGB surgery and who then follow one of the 6 post-surgical trajectories. Baseline demographic characteristics are taken from a Canadian study of wait-listed, medically-managed and bariatric surgical patients in Alberta [14]. Using the data of the surgical cohort, the hypothetical patients considered here were 87% female, 43.5 ± 9.5 years of age with body mass index (BMI) of 46.2 ± 7.2 kg/m^2^. Baseline co-morbidities were 45, 61 and 60% for T2D, HTN and DLP respectively (Table 1).

**Table 1:** Model parameters

Parameter	Reference	Base case	Notes
Age	Padwal et al. 2014 [10]	43.5 ± 9.5 years	Use population demographics of a Canadian surgical cohort (150 patients, Alberta)
BMI		46.2 ± 7.4 kg/m^2^	
Female		87.3% ± 2.7%	
T2D baseline		44.7% ± 4.1%	
HTN baseline		61.3% ± 4.0%	
DLP baseline		60.0% ± 4.0%	
Cohort size	N/A	100 patients	Example cohort
Provincial RYGB surgical volume	CIHI report [4]	Ontario 2380	Most recent data available for the provinces with top 3 RYGB volumes in year ending 2014
		Quebec 310	
		Alberta 260	
Discount rate	CADTH guidelines [15]	1.5%	4th edition guidelines
Cost T2D, year 1	Rosella et al. 2016 [16]	Male: $4061 ± $609	Ontario Base value uncertainty taken as ±15%
		Female: $4017 ± $603	
Cost T2D, year 2+	Rosella et al. 2016 [16]	Male: $828 ± $123	Ontario Base value average costs years 2–8 in study
		Female: $1023 ± $124	
Cost HTN	Weaver et al. 2015 [17]	$2163 ± $227	Canada wide
Cost DLP	Conly et al. 2011 [18]	$79 ± $8	Alberta Final value includes only laboratory costs for patients on statins minus costs for patient time and travel

Costs from reported currency year were inflated to 2018 Canadian dollars using Statistics Canada consumer price index data for health services and products. CADTH, Canadian Agency for Drugs and Technologies in Health; CIHI, Canadian Institute for Health Information; DLP, dyslipidaemia; HTN, hypertension; NA, not applicable; RYGB, Roux-en-Y gastric bypass; T2D, type 2 diabetes mellitus

### Outcomes of weight loss trajectory

Non-linear regression models were investigated for fitting to the observed patient trajectory group data of Courcoulas et al. [10] Group labels in the present study correspond to those reported there, with broadly increasing weight loss from the poorest in group 1 (G1) to the best outcomes in group 6 (G6) over the post-RYGB period. Model fits were determined separately for each trajectory group using CurveExpert Professional version 2.6 and the best model for each was selected based on a score derived in part from the Akaike Information Coefficient adjusted for small sample sizes (AICC). Yield density models were found to be the most suitable for the extremes (G1 and G6) while the intermediate weight loss trajectories were best fit with reciprocal quadratic models (G2 to G5). The modelled total weight loss (TWL) percentage was applied to the hypothetical cohort baseline demographics to calculate BMI over the 10 years post-RYGB.

### Co-morbidity evolution

After bariatric surgery, many factors may contribute to the resolution of co-morbidities in patients with the disease at baseline or development of new onset disease in patients without. In the absence of detailed data, the present study estimated co-morbidity evolution by correlating the trajectory group outcomes reported by Courcoulas et al. [10] with the available, reported group demographic data over time post-surgery (age and BMI). Resolution data for each co-morbidity were reported for each trajectory group at time points of 6 months, and years 1, 2, 3, 4, 5 and 7 post-surgery. Linear regression models were fit to associate patient post-surgical age and BMI with expected resolution; more complex models were not used to reduce the risk of over fitting. Models for each co-morbidity were determined to estimate resolution as a function of post-RYGB age and BMI in patients of the hypothetical cohort who had the disease at baseline.

Incident disease after RYGB has been reported to be low [10, 19]. The incident data of Courcoulas et al. [10] were not stratified by trajectory group, meaning only total numbers of new cases by post-surgical timepoint were available for modelling. As above, in the absence of further data, a parsimonious approach was used with linear models to predict new onset cases in patients of the hypothetical cohort who did not have disease at baseline as a function of the overall cohort BMI at each post-surgical timepoint.

For both the remission and new onset groups, patients are assumed to remain within their respective groups over the time horizon. Patients with the co-morbidity at baseline who achieve remission therefore remain in that group and are not added to the group at risk for new onset. Similarly, new onset cases cannot achieve remission, as there were no data to inform accurate estimates of this movement.

### Cost analysis

The overall prevalence of each co-morbidity at 1-year intervals post-RYGB was calculated as the sum of non-resolved and new onset cases. Total patient-years of treatment of co-morbidities were determined over the 10-year time horizon for each co-morbidity using annual treatment costs in the Canadian public health care system (Table 1). A study from Canada has suggested that patient treatment costs may increase according to overweight and obesity (relative to normal weight) independently of presence of co-morbidities [20]. Since the present analysis calculates BMI over time after surgery for each trajectory group, a scaling factor was calculated for cost increase as a function of BMI. Curve-fitting (including linear regression) was performed yielding a reciprocal linear relationship as the most suitable to model the annual cost-scaling factor as a function of BMI.

Input costs are shown in Table 1. All costs are for public payers in the Canadian context. Costs for T2D and HTN thus include physician and hospital treatments, while those for DLP are the public costs of additional lab work for patients receiving statins. An annual discount rate of 1.5% was applied across the 10-year time horizon.

### Sensitivity analysis

The robustness of the base case cohort was assessed through probabilistic sensitivity analysis with 10,000 iterations. For each iteration, parameters were sampled using the normal distribution (age, initial BMI, proportion of female patients, the proportion of patients at baseline with T2D, HTN or DLP, and all cost parameters) according to the mean and standard deviation for each. The 10-year total patient-years of treatment and costs were calculated, from which 95% credibility intervals (95% CrIs) were determined. Calculations were performed using the R statistical programming language version 3.6 (R Project for Statistical Computing).

### Scenario analyses

The primary analysis considered a hypothetical group of 100 patients following each of the 6 separate trajectories. It is expected that in real world practice, there will be a distribution of patients among different weight loss trajectories. To estimate outcomes in co-morbidity burden and corresponding costs in a more representative cohort, scenario analyses were generated of 100 patient cohorts in which different proportions of patients follow the different trajectories (Table 2). In the base case scenario, taken as standard care, patients are distributed according to the proportions reported in Courcoulas et al. [10] with, for example, 13.3% of patients in the best (G6) post-RYGB weight loss trajectory and 4.8% in the lowest (G1). The first improvement scenario considers a case where all patients are improved to the next best trajectory according to area under the curve for 10-year weight loss. Patients in the poorest G1 therefore are moved to G2, G2 to G3 and so on. Patients in the best (G6) trajectory remain there and are not considered to improve further. In the second improvement scenario, patients are redistributed among the top 3 trajectories (G4, G5 and G6). Co-morbidity patient-years of treatment and associated costs were calculated for the entire 100-patient cohort according to each scenario. Differences between the improved and standard care scenarios were further assessed in the context of reported RYGB surgical volume for the top 3 Canadian provinces performing the procedure in 2014.

**Table 2:** Patient distribution by weight loss trajectory for scenario analyses

Group	Standard Care	Broad Improvement	Top 3 Trajectories
G6	13.3%	39.6%	29.2%
G4	26.3%	6.1%	57.7%
G5	6.1%	27.8%	13.4%
G3	27.8%	21.6%	0.0%
G2	21.6%	4.8%	0.0%
G1	4.8%	0.0%	0.0%

Post-gastric bypass weight loss trajectories (G1 to G6 as defined in reference [10]) are shown ordered from greatest to lowest 10-year total weight loss, and percentages indicate the distribution of patients according to scenario. The standard care represents the base case; in broad improvement, all patients are shifted up to the next best trajectory (those in the best trajectory, group 6, remain there); in the final scenario, all patients are redistributed among the top 3 trajectories by total 10-year weight loss

### Statistical analysis

Values are reported as medians and 95% CrIs determined from the 10,000 replicates. Differences may be considered significant for 95% CrIs that do not include zero (which would indicate that 95% of simulation results include both positive and negative differences). However, intervals are presented to allow readers individually to determine the relevance of the reported estimates according to local context, independently of statistical determination of significance.

## Results

The present analysis made extensive use of statistical models to predict outcomes from observed data. A primary consideration is therefore the appropriateness and quality of those fits as fundamental to how reasonable derived outcome estimates may be. As described (see Methods) a score was used that is based on multiple factors to determine the most appropriate model for each fit. One measure of fit quality is the coefficient of determination as it provides an estimate of the proportion of the variance in the observed outcomes that is explained by the fitted model. Results in the present study suggest that the models used generally demonstrate high quality (by this metric) with most fits explaining around 80% or more of the variance in the output variable of interest (Table 3). One exception is the modelling of DLP remission, which demonstrated the poorest fit. Nevertheless, in the absence of further data and to prevent over-fitting, this model was used subsequently for further analyses. It should be noted that these results indicate only the correlation between the input variables (such as age and BMI) with predicted outputs (such as co-morbidity resolution rates) and therefore make no assertion regarding other factors that may contribute to the outcome.

**Table 3:** Model fitting results

Outcome	Model type	Coefficient of determination
Group 1 TWL	Yield-density	95.7%
Group 2 TWL	Reciprocal quadratic	98.9%
Group 3 TWL	Reciprocal quadratic	99.3%
Group 4 TWL	Reciprocal quadratic	99.4%
Group 5 TWL	Reciprocal quadratic	99.0%
Group 6 TWL	Yield-density	99.9%
T2D resolution	Linear, 2 independent variables	80.8%
T2D incidence	Linear, 1 independent variable	79.4%
HTN resolution	Linear, 2 independent variables	84.3%
HTN incidence	Linear, 1 independent variable	88.6%
DLP resolution	Linear, 2 independent variables	42.4%
DLP incidence	Linear, 1 independent variable	92.6%
BMI cost factor	Reciprocal linear	97.8%

Assessment of fitting quality of various models used in the present study. BMI, body mass index; DLP, dyslipidaemia; HTN, hypertension; T2D, type 2 diabetes mellitus; TWL, total weight loss

**Fig. 2:**
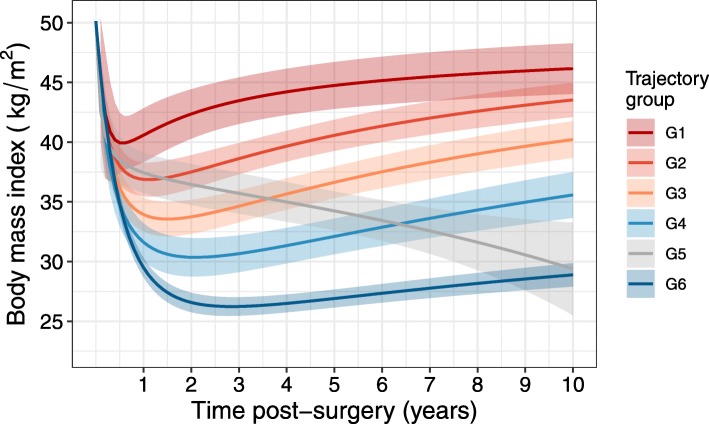
Modelling of post-surgical body mass index. Total weight loss (TWL) data were modelled from fitting of patient observational data stratified into trajectory groups as presented in the report by Courcoulas et al. [10] The post-surgical TWL fits were applied to a cohort with a baseline body mass index of 46.2 ± 7.4 kg/m^2^ to determine the BMI of each trajectory over the 10 years post-surgery. Bands correspond to 95% confidence intervals of prediction around the fitted lines

Results of the fitting of post-surgical BMI by trajectory group are shown in Fig. 2. Most groups (G1, G2, G3, G4, G6) exhibit a similar overall shape, with post-surgical weight loss reaching a nadir between 6 months (G1) and 2.5 years (G6), followed by varying degrees of weight regain. G5 is an exception, in which patients had low 6-month weight loss similar to groups G1 through G3, but subsequently the patients embark on pronounced and maintained weight loss with no evidence of weight regain.

**Fig. 3:**
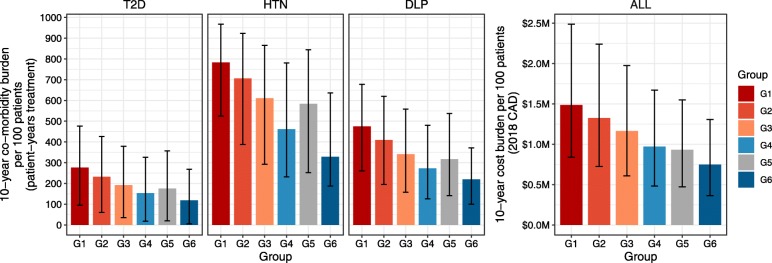
Total 10-year co-morbidity treatment and costs by post-surgical weight loss trajectory group. Results shown indicate the total 10-year burden in patient-years of treatment of co-morbidities and corresponding costs (millions, 2018 Canadian dollars) associated with a 100-patient cohort’s following each of the weight loss trajectories (see Fig. 2) after Roux-en-Y gastric bypass surgery. Bars indicate median values and error ranges are 95% CrIs from 10,000 iterations in sensitivity analyses. CAD, Canadian dollars; DLP, dyslipidaemia; HTN, hypertension; bypass; T2D, type 2 diabetes mellitus

Models of BMI and time after surgery (converted to patient age) were used to calculate total 10-year outcome burden by weight loss trajectory of T2D, HTN and DLP co-morbidities as patient-years of treatment, and as total overall costs (Fig. 3). As expected, co-morbidity burden and costs associated with weight loss trajectory in general decrease as the trajectory improved with greater 10-year weight loss and correspondingly fewer patient-years of treatment of co-morbidities. The uncertainty ranges (95% CrIs) among outcomes associated with different weight loss trajectories demonstrate a high degree of overlap, however results are highly correlated, meaning values towards the upper end of the uncertainty interval in one trajectory will pair with similarly high values towards the upper end of the interval for another trajectory. This effect is demonstrated through calculation of percent difference in burden calculated pairwise between trajectory groups (Table 4). The percent difference is calculated independently for each of the 10,000 iterations of the baseline sensitivity analysis and the resulting 95% CrIs show that in every instance of shifting a 100-patient cohort from a poorer to a better weight loss trajectory, there is a proportional reduction in 10-year cost burden that does not include instances of cost increase (intervals exclude 0). The most extreme improvement, from group G1 to G6 is associated with a change of − 50% (95% CrI − 60% to − 41%) of treatment costs of co-morbidities relative to G1 costs. An intermediate change from G2 to G4 was associated with a − 27% change (95% CrI − 35% to − 23%) relative to the G2 costs. The smallest observed difference, a shift from G4 to G5 was also associated with an interval that did not overlap with 0 (median − 4, 95% CrI − 8% to − 2%).

**Fig. 4:**
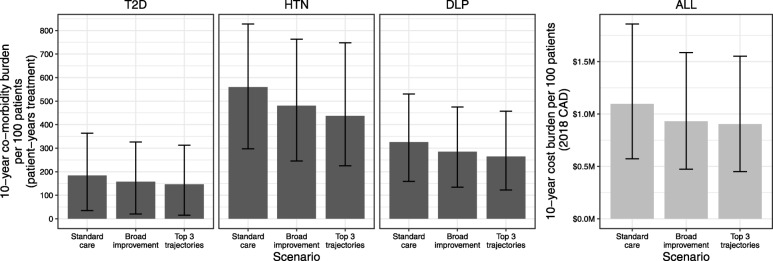
Total 10-year co-morbidity treatment and costs by scenario analysis of mixed-weight loss trajectory cohorts. Results are shown for burden (co-morbidity patient-years of treatment and corresponding costs in millions of 2018 Canadian dollars) associated with mixed weight trajectory cohorts. The distributions of patients among the trajectories are listed in Table 2. Standard care represents the case where patients are distributed among all 6 trajectories, broad improvement has all patients on trajectories lower than the best moved up to the next best trajectory, and the top 3 trajectories scenario redistributes patients among the top 3 best trajectories. Bars indicate medians and error ranges 95% CrIs. CAD, Canadian dollars; DLP, dyslipidaemia; HTN, hypertension; T2D, type 2 diabetes mellitus

**Table 4:** Proportional difference in total 10-year costs according to post-surgical weight loss trajectory improvement

Group	G1	G2	G3	G4	G5	G6
G1	0.0% [0.0, 0.0%]					
G2	−11.0% [− 14.1, −8.8%]	0.0% [0.0, 0.0%]				
G3	−22.2% [−28.0, − 18.3%]	−12.6% [− 16.3, − 10.3%]	0.0% [0.0, 0.0%]			
G4	−35.3% [− 43.5, − 29.5%]	−27.3% [− 34.6, − 22.6%]	−16.8% [− 22.4, − 13.2%]	0.0% [0.0, 0.0%]		
G5	−38.1% [−45.3, − 32.7%]	−30.5% [− 36.7, − 25.7%]	−20.5% [− 24.9, − 15.9%]	−3.8% [− 7.8, − 1.8%]	0.0% [0.0, 0.0%]	
G6	−50.2% [− 59.9, − 41.2%]	−44.0% [− 53.7, − 34.9%]	−36.0% [− 45.6, − 26.6%]	−23.0% [− 31.4, − 15.2%]	−19.3% [− 28.6, − 12.3%]	0.0% [0.0, 0.0%]

Values shown indicate the percentage change in total 10-year costs for a cohort of 100 patients who experience improvement in weight loss trajectory (G1 poorest, G6 greatest weight loss). Starting trajectories are across the top and destination trajectories vertically. An extreme improvement of 100 patients from G1 to G6 is therefore estimated to be associated with a 50% reduction in costs of treatment of co-morbidities, while an intermediate improvement from G2 to G4 is estimated to be associated with 27% lower costs of co-morbidity treatment over 10 years. Co-morbidities are restricted to the three of the present study (type 2 diabetes mellitus, hypertension and dyslipidaemia)

It is expected in real-world practice that cohorts of patients will exhibit a range of weight loss trajectories after surgery. The scenario analyses investigated the potential effect of varying improvements in weight loss trajectory for patients in such a mixed cohort (Fig. 4). As for the homogenous 100-patient cohorts by trajectory, determination of patient-years of treatment of co-morbidities and corresponding costs showed decreases according to increasing improvements in patient outcomes. The 95% CrIs again overlap considerably for the overall 10-year total outcomes, but differences calculated by iteration reveal intervals that do not include zero. These results (difference in burden between improved scenarios and standard care) were scaled to the context of the three provinces with the greatest RYGB surgical volume, namely Ontario, Quebec and Alberta. Estimates show that the intervals around the medians for treatment of co-morbidities (Table 5) and for the costs by co-morbidity and overall (Table 6) are exclusive of zero.

**Table 5:** Additional burden in total 10-year co-morbidity for standard care relative to scenarios of improved care

Province (volume)	Scenario	Type 2 diabetes mellitus	Hypertension	Dyslipidaemia
Ontario (2380)	Broad improvement	602 [261; 958]	1710 [1004; 2190]	966 [396; 1398]
	Top 3 trajectories	952 [313; 1296]	2628 [1231; 3248]	1612 [471; 1936]
Quebec (310)	Broad improvement	78 [34; 125]	223 [131; 285]	126 [52; 182]
	Top 3 trajectories	124 [41; 169]	342 [160; 423]	210 [61; 252]
Alberta (260)	Broad improvement	66 [29; 105]	187 [110; 239]	106 [43; 153]
	Top 3 trajectories	104 [34; 142]	287 [134; 355]	176 [51; 211]

Values shown represent the additional burden of treatment of co-morbidities (patient-years) associated with the standard care pathway relative to scenarios of improved care after Roux-en-Y gastric bypass surgery. Results were scaled according to Roux-en-Y gastric bypass volume in the top 3 Canadian provinces in the year ending in 2014 [4].

**Table 6:** Additional total 10-year costs for standard care relative to scenarios of improved care

Province (volume)e	Scenario	Type 2 diabetes mellitus	Hypertension	Dyslipidaemia	Total
Ontario (2380)	Broad improvement	$636,000 [$255,000; $1,302,000]	$3,226,000 [$1,837,000; $5,376,000]	$70,000 [$36,000; $129,000]	$3,938,000 [$2,185,000; $6,709,000]
	Top 3 trajectories	$760,000 [$309,000; $1,476,000]	$3,745,000 [$2,219,000; $6,025,000]	$84,000 [$42,000; $145,000]	$4,599,000 [$2,627,000; $7,561,000]
Quebec (310)	Broad improvement	$83,000 [$33,000; $170,000]	$420,000 [$239,000; $700,000]	$9000 [$5000; $17,000]	$513,000 [$285,000; $874,000]
	Top 3 trajectories	$99,000 [$40,000; $192,000]	$488,000 [$289,000; $785,000]	$11,000 [$5000; $19,000]	$599,000 [$342,000; $985,000]
Alberta (260)	Broad improvement	$69,000 [$28,000; $142,000]	$352,000 [$201,000; $587,000]	$8000 [$4000; $14,000]	$430,000 [$239,000; $733,000]
	Top 3 trajectories	$83,000 [$34,000; $161,000]	$409,000 [$242,000; $658,000]	$9000 [$5000; $16,000]	$502,000 [$287,000; $826,000]

Values shown represent the additional costs (2018 Canadian dollars, rounded to the nearest $1000) associated with the standard care pathway relative to scenarios of improved care after Roux-en-Y gastric bypass surgery. Results were scaled according to Roux-en-Y gastric bypass volume in the top 3 Canadian provinces in the year ending in 2014 [4].

## Discussion

After bariatric surgery, patients experience varying degrees of weight loss. The aim of the present study is to provide a first estimate of the impact of a variety of weight trajectories on patient outcomes and treatment costs. While many studies may assess entire surgical cohorts as single arm studies, or comparisons against other treatments (surgical or non-surgical) for weight loss and co-morbidity effects, this analysis sought to examine differences among patients who received the same surgery and were at the same risk of surgery-related complications and associated costs, but by some means achieved different weight trajectories.

Our analysis suggests that better post-RYGB weight trajectories are associated with a considerably lower burden of patient-years of treatment of co-morbidities (T2D, HTN and DLP) and the corresponding costs. The improvement of 100 patients by even one trajectory to the next best set of weight loss outcomes was associated with median 12.5% lower 10-year costs (G2 to G3). Consideration of a potential real-world cohort of patients experiencing a mixture of weight trajectories after RYGB also suggests lower costs associated with broad or targeted improvements in the distribution of patient trajectories. Among the top three provinces by 2014 RYGB volume, the potential cost differences ranged from $430,000 for broad improvement in Alberta to $4.6 million in Ontario if all patients achieve the top 3 weight trajectories.

As a first detailed investigation of co-morbidity outcomes and cost impact due to variations in weight trajectories after RYGB surgery, the study is not without limitations. The availability of data in the North American setting for post-surgical co-morbidity evolution by weight trajectory permitted the derivation of descriptive models after RYGB, but it must be assumed that the results from this single American cohort also apply to the Canadian setting. Evaluation of model fits suggests that the models selected, and the approach performed well, but these predictions remain an estimation in the absence of true real-world clinical data. Also, the reported post-surgical co-morbidity incidence was not stratified by weight trajectory [10]. If the new cases demonstrated a tendency towards developing in patients with a poorer weight trajectory, the incidence calculated here would represent an overestimate for the weight outcomes that demonstrate higher long-term weight loss.

The cost analysis is potentially a conservative estimate of the true costs associated with weight trajectory outcome. Due in part to availability of detailed data, only chronic conditions (T2D, HTN, and DLP) were included. Surgery also has an impact on other cost-attributable obesity-related conditions such as cardiovascular disease risk (including myocardial infarction, stroke or heart failure), [21] osteoarthritis requiring joint replacement surgery [22] and non-alcoholic fatty liver disease [23]. Bariatric surgery has been shown for these conditions to have a positive effect in reducing their prevalence, and one might thus expect considerable corresponding differences in treatment costs, but in the absence of further data, no reasonable estimate could be determined. The costs used were also drawn from individual provinces and may therefore not reflect local differences in province-specific costs and treatments. This limitation is in part addressed by the determination of the associated patient-years of co-morbidity treatment, which can be readily converted to costs according to jurisdiction, and further expression of differences as percentage changes. It should be noted that cost differences between improved weight trajectories or improved mixed trajectory cohort scenarios represent reductions in burden related to the analysed co-morbidities. Whether the estimates result in cost savings will be affected by whatever interventions may be required to achieve the improved weight trajectories.

There are multiple factors influencing weight loss outcomes after surgery. Among them, genetics in the form of single nucleotide polymorphisms (SNPs), [11] sex or gender and race have been found to influence weight loss outcomes [10]. Future studies may identify ways that patient care can be optimised for patients with these different characteristics to improve outcomes. Until such time, providers can focus on other aspects of care that have been shown to be associated with improved weight loss. These include patient behaviours in eating and exercise, [24] better follow-up attendance [13, 24, 25] as well as achieving better outcomes at specialist bariatric care centres over non-specialist care [26]. Additional interventions may also improve trajectories, such as increasing the role of dietitians [27] and psychologists for follow-up behavioural therapy, [28] or physical therapy and exercise programs may encourage more physical activity [29]. The present analysis serves as an estimate of what is at stake for providers in terms of patient outcomes and associated costs should post-bariatric surgery trajectories not be optimised. With this information, informed decisions can be made regarding investment in strategies targeted towards modifiable factors of patient behaviour and engagement to achieve improved patient outcomes.

## Conclusions

After bariatric surgery, including RYGB, patients experience a variety of weight trajectories. Even after the same surgery, patients may achieve good trajectories with sustained weight loss, or poorer outcomes with less weight loss and considerable weight regain. The trajectory can be associated with successful co-morbidity resolution, and patient risk of new onset disease. Having a poor weight trajectory increases patient time with co-morbidities and is associated with increased costs of care. Improving weight trajectories after RYGB was estimated to be associated with lower costs of co-morbidity treatment: up to 50% lower in the extreme of shifting patients from the poorest trajectory to the best, 27% lower for an intermediate improvement. System-wide improvements at the provincial level were associated with $430,000 (Alberta) to $4.6 million (Ontario) lower co-morbidity treatment costs. This analysis is an initial estimate of burden, but its results provide justification for debate on whether investment of resources is required to optimise patient weight trajectories, realise better outcomes among patient co-morbidities, and ultimately improve and lower the cost of healthcare provision for these common obesity-related chronic conditions.

## Data Availability

The datasets used and/or analysed during the current study are available from the corresponding author on reasonable request.

## Abbreviations

BMI: Body mass index
DLP: Dyslipidaemia
HTN: Hypertension
RYGB: Roux-en-y gastric bypass surgery
T2D: Type 2 diabetes mellitus
TWL: Total weight loss
